# A Serious Video Game to Increase Fruit and Vegetable Consumption Among Elementary Aged Youth (Squire’s Quest! II): Rationale, Design, and Methods

**DOI:** 10.2196/resprot.2348

**Published:** 2012-11-21

**Authors:** Debbe Thompson, Riddhi Bhatt, Melanie Lazarus, Karen Cullen, Janice Baranowski, Tom Baranowski

**Affiliations:** 1 USDA/ARS Children's Nutrition Research CenterDepartment of PediatricsBaylor College of MedicineHouston, TXUnited States; 2 Department of PediatricsBaylor College of MedicineHouston, TXUnited States; 3 St. Luke's Episcopal HospitalTexas Heart Institute6770 Bertner AvenueHouston, TXUnited States

**Keywords:** video game, nutrition, fruit, vegetable, children, intervention, action implementation intention, coping implementation intention, goal setting

## Abstract

**Background:**

Youths eat fewer fruits and vegetables than recommended. Effective methods are needed to increase and maintain their fruit and vegetable consumption. Goal setting has been an effective behavior change procedure among adults, but has had limited effectiveness among youths. Implementation intentions are specific plans to facilitate goal attainment. Redefining goal setting to include implementation intentions may be an effective way to increase effectiveness. Video games offer a controlled venue for conducting behavioral research and testing hypotheses to identify mechanisms of effect.

**Objective:**

This report describes the protocol that guided the design and evaluation of Squire’s Quest! II, a video game aimed to increase child fruit and vegetable consumption.

**Methods:**

Squire’s Quest! II is a 10-episode videogame promoting fruit and vegetable consumption to 4th and 5th grade children (approximately 9-11 year old youths). A four group randomized design (n=400 parent/child dyads) was used to systematically test the effect of two types of implementation intentions (action, coping) on fruit and vegetable goal attainment and consumption of 4th and 5th graders. Data collection occurred at baseline, immediately post game-play, and 3 months later. Child was the unit of assignment. Three dietary recalls were collected at each data collection period by trained interviewers using the Nutrient Data System for Research (NDSR 2009). Psychosocial and process data were also collected.

**Results:**

To our knowledge, this is the first research to explore the effect of implementation intentions on child fruit and vegetable goal attainment and consumption.

**Conclusions:**

This intervention will contribute valuable information regarding whether implementation intentions are effective with elementary age children.

**Trial Registration:**

ClinicalTrials.gov NCT01004094

## Introduction

Consuming adequate amounts of fruit and vegetables (FV) is part of a healthy lifestyle [1] and has been associated with decreased risk of chronic diseases such as certain cancers, cardiovascular disease, stroke, and diabetes [2]. National guidelines recommend that 9-13 year old youths consume 7-11 servings of FV each day, based on calorie needs [3]. However, less than 4% of children meet the minimum guideline, and fewer than 20% consume at least five servings each day [4]. Since adolescent dietary behaviors track into young adulthood [5], increasing and maintaining youths’ FV consumption prior to adolescence could have substantial and sustained public health significance.

Video games are popular among youths [6]. Many youths have ready-access to cell phones, game consoles, and computers on which video games can be played [6]. High-speed home Internet access has increased among households with youths [6]. Therefore, online video games may be a familiar and convenient method to reach youths with health-enhancing programs. They also offer a mechanism for ensuring consistent intervention delivery, thus controlling for potential lack of fidelity to standardized content and implementation procedures potentially introduced by live instructors [7].

Serious video games, ie, video games designed to entertain as well as achieve change of some type [8], is an emerging genre [9], with some reported success at changing health behaviors [8]. Squire’s Quest! increased FV consumption among 4^th^ grade children [10]; however post-study consumption was still well-below recommended levels, suggesting additional investigation was needed. Secondary analyses revealed that goal setting was weakly related to goal attainment and FV consumption [11], suggesting that enhancing the goal setting component of Squire’s Quest! may offer a mechanism to further enhance participants’ FV consumption.

Implementation intentions are specific plans that identify how to achieve a goal [12]. They can take two forms: (1) action intentions, a specific plan of how a goal will be attained (ie, what, when, who), and (2) coping intentions, an if/then plan that identifies what solution an individual will enact if a specific obstacle or problem is encountered [12]. When forming an implementation intention, an individual determines in advance how to meet a goal by examining possible situations and selecting the ones most likely to lead to goal attainment [12]. Environmental cues rather than conscious thought trigger a goal-directed response, thereby automating behavior and increasing the likelihood the goal will be attained [13]. Implementation intentions have enhanced goal attainment across a variety of adult health behaviors [14-18]. There is some evidence they may be effective with youths - ie, adolescents who formed implementation intentions prior to initiating an academic goal were more likely to achieve the goal than adolescents who did not form an implementation intention [19].

Long-term behavior change is the ultimate goal for behavioral interventions [20]. However, little research has specifically addressed the issue of maintaining dietary behavior change [21]. While some dietary change interventions have occurred over a two-year period [22], no conceptual distinction was made between initiation and maintenance of change. Desired changes in targeted dietary behavior in the intervention group relative to the control group have shown mixed effects [23]. Among adults, outcome expectancy was conceptualized to be related to behavior initiation, while perceived satisfaction with behavioral change was conceptualized to be associated with maintenance [20]. Satisfaction is a continual assessment of whether the “benefits” of change were worth the effort to make and/or continue the change. Since high expectations may be more likely to lead to dissatisfaction, high outcome expectancies were hypothesized to be associated with greater behavioral initiation, but lower maintenance, while more modest expectations, were hypothesized to be associated with lower behavioral initiation, but greater maintenance [20].

Squire’s Quest! II: Saving the Kingdom of Fivealot (SQ!2) is a 10-episode online video game designed to increase FV consumption among 4^th^ and 5^th^ grade children (roughly 9-11 year old youths). It is an updated and enhanced version of an earlier video game, Squire’s Quest!, evaluated in 1999 and 2000 [10]. SQ!2 was supported by a parent component which included electronic newsletters and access to a parent-only website. The primary aim of SQ!2 was to test the effect of implementation intentions on FV goal attainment and consumption in pre-adolescents. An exploratory aim was to examine factors associated with maintenance of consumption. The protocol was approved by the institutional review board of the Baylor College of Medicine (H-18488) and registered with ClinicalTrials.gov (NCT01004094). This report describes the study protocol that guided the design and evaluation of the SQ!2 randomized controlled trial.

## Methods

### Study Design

**Figure 1:**
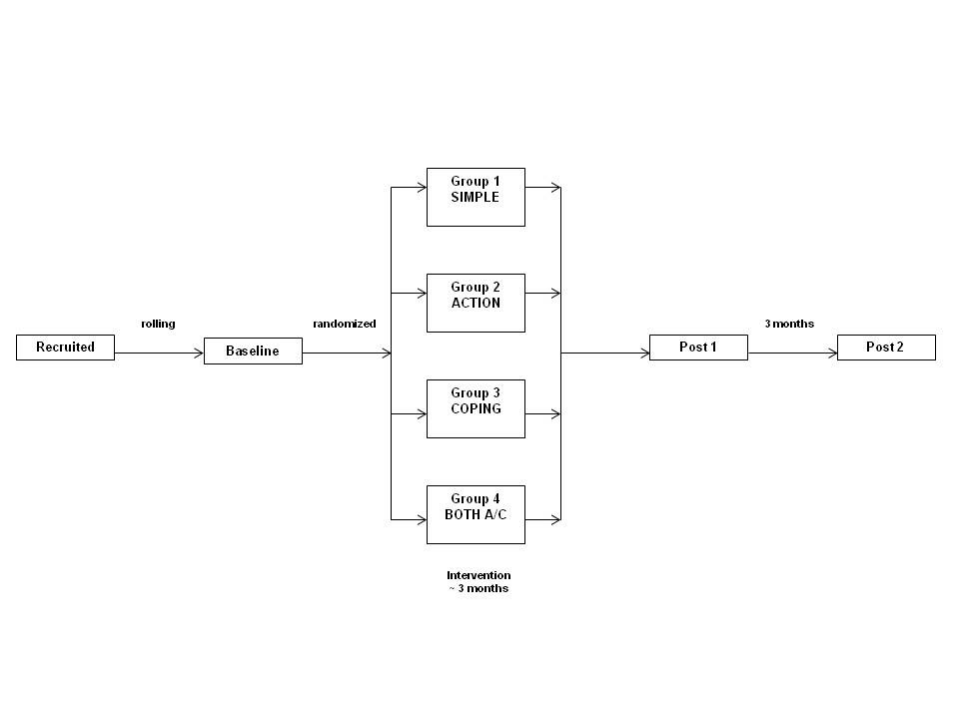
Research design.

This evaluation used a four-group, randomized design, with three data collection periods (baseline, post 1, post 2). Following baseline assessment, children were randomized to one of four groups: goal setting only (simple), goal setting + action intentions (action), goal setting + coping intentions (coping), or goal setting + action and coping intentions (both). Youths had up to three months to play all 10 episodes of the video game, where the appropriate goal-setting/implementation intentions were embedded in four versions of the game. Post 1 data collection occurred immediately upon completion of the 10 episodes or approximately 3 months after beginning game-play, whichever occurred first. Post 2 data collection occurred approximately 3 months after post 1 (Figure 1). The study was conducted from November 2009 through March 2011.

### Participants

Eligibility criteria for participants included: (1) a child in the 4^th^ or 5^th^ grade at time of enrollment, (2) fluent in English, (3) had access to a computer with high speed Internet, and (4) had a parent (or legal guardian) fluent in English or Spanish who was willing to participate in the study. Recruitment methods included standard procedures (ie, flyers and attendance at community events) and the volunteer database at the Children’s Nutrition Research Center. Prior to participation, eligible parents and children provided written informed consent and child assent. The child was the unit of randomization.

### Sample Size and Power

Sample size requirements were based on a power analysis (the number of participants needed to find an actual group difference)  with FV as the primary outcome variable. A repeated measures analyses of variance with a 4-group design and 3-measurment periods was assumed. Allowing for a 30% attrition rate, a sample of 400 parent/child dyads (100 per group) provided adequate power (>80%) to detect a small effect size (ES=0.17) using a standard deviation of 1.5 from the original Squire’s Quest! and a two-sided alternative with type I error rate of 0.05. This effect size (ES=0.17) translates to a detectable 0.51 or greater serving (ie, a half of a serving per day) group difference.

### Sample Characteristics

Four hundred multi-ethnic parent/child dyads were enrolled in the study. Fifty three percent of children were girls, while 47% were boys; racial/ethnic distribution was 37% White, 27% Hispanic, 26% Black, and 10% Other. Most parents were female (96%), and parent racial/ethnic distribution was similar to that of the children (41% White; 26% Hispanic; 26% Black; 7% Other).

### Setting

Parents and children participated in separate intervention and data collection activities electronically (Internet or telephone) from locations of their choice (home or community). There were no face-to-face sessions.

### Intervention

**Figure 2:**
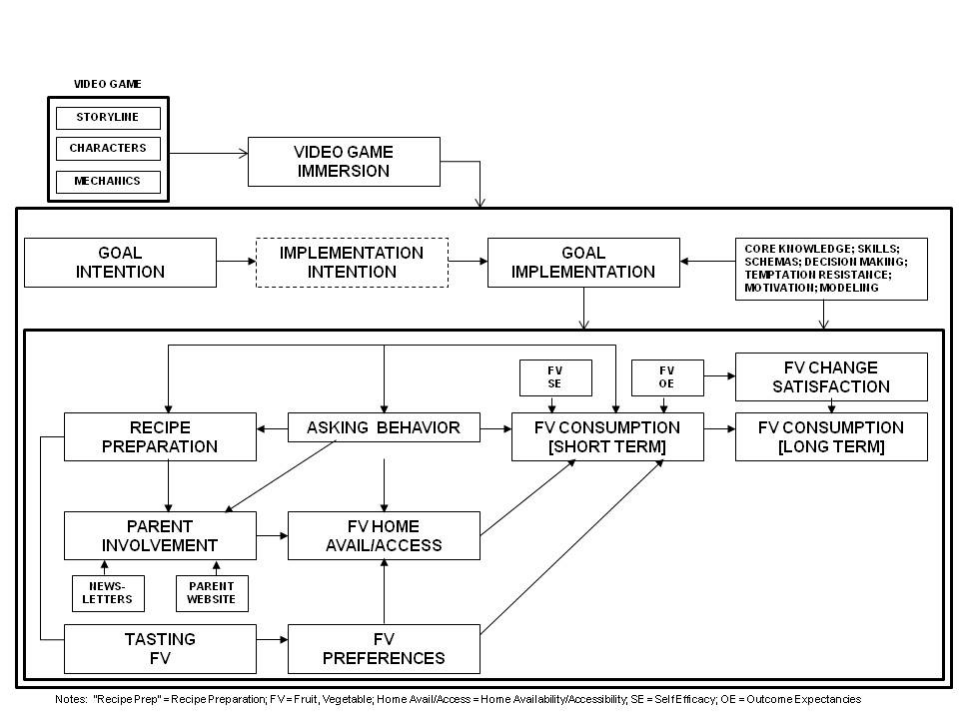
Conceptual model.

The intervention had both child and parent components. The child played a 10-episode online video game, while the parent received electronic newsletters and access to a parent website which was updated with new information10 times, corresponding to the 10-episode video game. The intervention was guided by a theoretical framework that incorporated social cognitive (behavioral and environmental factors) [24], self determination (motivation) [25], the elaboration likelihood model (information processing) [26], behavioral inoculation (resistance to temptation) [27], and maintenance theories (long-term behavior change) [20]. The conceptual model that guided the intervention is presented in Figure 2. It provides an overview of how the intervention promotes short and long term FV consumption. The story, characters, and game mechanics (eg, interactivity) promote immersion, which increases exposure to the behavior change components (ie, program dose). Setting a goal and/or creating an implementation intention, developing core knowledge and skills, and engaging in key behavioral procedures (eg, schemas, decision making, resisting temptation, motivation, and character modeling) contribute to FV self efficacy and outcome expectations. FV self efficacy and outcome expectations influence short and long term FV consumption (maintenance). Outcome expectations also influence satisfaction, which influences long term consumption. In addition, the parent component (newsletters, website), coupled with child asking behaviors and recipe preparation, influence parent involvement; parent involvement, FV tasting, and FV preferences influence FV home availability and accessibility and short and long-term FV consumption.

### Child Component: Video Game

#### Development

Multi-ethnic 4^th^ and 5^th^ grade children provided information for the development and initial testing of the video game. Children (n=15) provided feedback on behavior change components, the storyline, and the characters. Children (n=10) also participated in alpha and beta testing sessions. An experienced 4^th^ grade teacher reviewed materials to assess developmental appropriateness and comprehension of key components. Children and their parents (n=20 dyads) also participated in a pilot study, which served as a final test for the intervention components, procedures, and data collection activities of the video game.

#### Storyline

The story, written by a professional writer, was designed to appeal to 4^th^ and 5^th^ grade youths (ie, humor appropriate for children). It integrated the behavior change components into the action, which, in turn, advanced the storyline. The “backstory” (ie, the history) provided context for the storyline.

#### Backstory

The medieval kingdom of Fivealot is a happy one; its prosperity is based on ancient knowledge of healthy nutrition and an abundance of good food, under the gentle guidance of King Brocwell and Queen Nutritia. Recently, Fivealot entered a truce with their underground-dwelling enemy, the Mog – or so they thought.

A nation of subterranean serpents and myopic moles, the Mog were jealous of Fivealot’s good fortune. Although Brocwell and Nutritia had offered to share their country’s bounty, the Mog who were raised on a diet of sweets and fried foods, stubbornly refused to change their ways. The Mog King Snake, Sssynster, instead insisted that Fivealot change *their* habits – and the struggle was on.

Discovering that their army of fattened moles and slovenly snakes were no match for the energetic knights of Fivealot, Sssynster withdrew, realizing he could never best Brocwell and Nutritia in open conflict. His best hope was treachery.

The King and Queen of Fivealot’s Head Chef, Supremo, was preparing to go to market one day. With the truce in effect, he suspected nothing – certainly not the tunnel dug into his kitchen by the Mog. Unbeknownst to Brocwell and Nutritia, Supremo simply disappeared, spirited away to the land of Mog.

Then came the second part of Sssynster’s plan: a double agent, Moledred, had been placed on the kitchen staff, waiting for the opportunity to advance to the Head of the Kitchen. With the majority of knights away spreading the bounty of Fivealot, Moldred’s mission was to prepare his specialty - fattening, unhealthy meals - for the King and Queen, and thus bring the kingdom to ruin. But what Sssynster and Moledred had not counted on was a Squire answering the King and Queen’s call for brave young men and women to train in the ways of Fivealot and become knights themselves. A Squire…like you.

#### Quest

To become a knight, the Squire had to acquire the coveted knowledge and skills of the Fivealot Knights. This is where the game began. In the quest for knighthood, the Squire had to overcome challenges. The challenges involved attaining “real world” FV consumption and recipe goals. As the Squire (ie, the participant) met their challenges, they earned badges and progressed in their journey towards knighthood. Because children typically consume well below the recommended level of daily FV [4], promoting a more modest goal appeared prudent; therefore, the goal promoted in the game was to ultimately consume at least 5 daily servings of FV.

#### Characters

**Figure 3:**
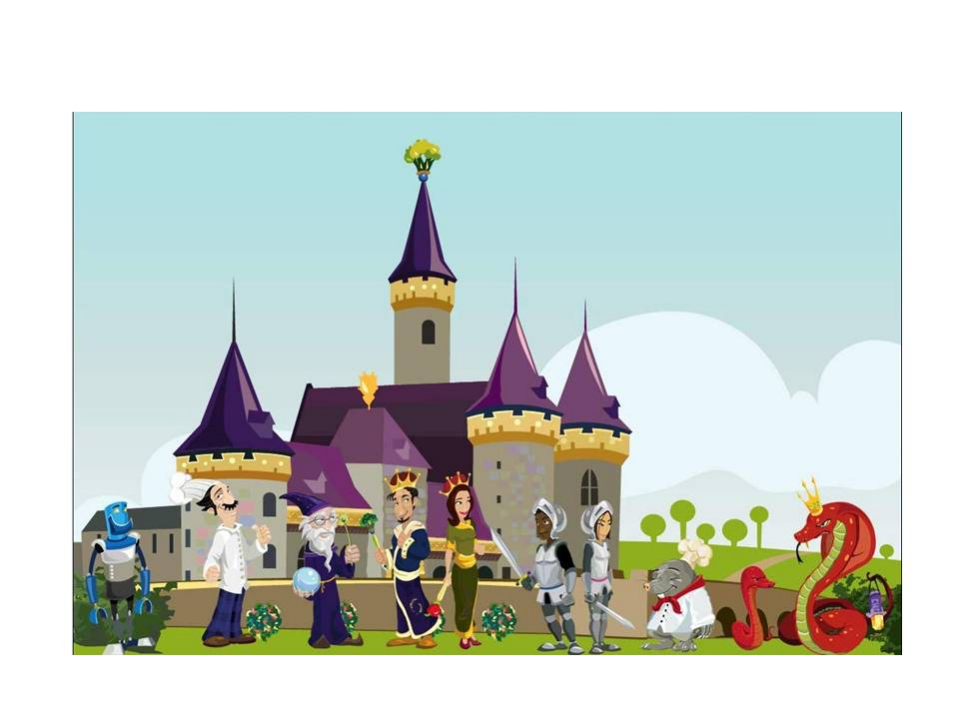
Characters.

The protagonists included six characters that were “human” in appearance (King Brocwell; Queen Nutritia; Merlin the Wizard; Knights Alex and Julie; Chef Supremo) and a robot (M.I.C.H.A.E.L.), who assisted with kitchen tasks. The antagonists were snakes (King Sssynster, a cobra; the snake army) and moles (Moledred, the imposter chef) (Figure 3).

#### Game Content and Structure

**Figure 4:**
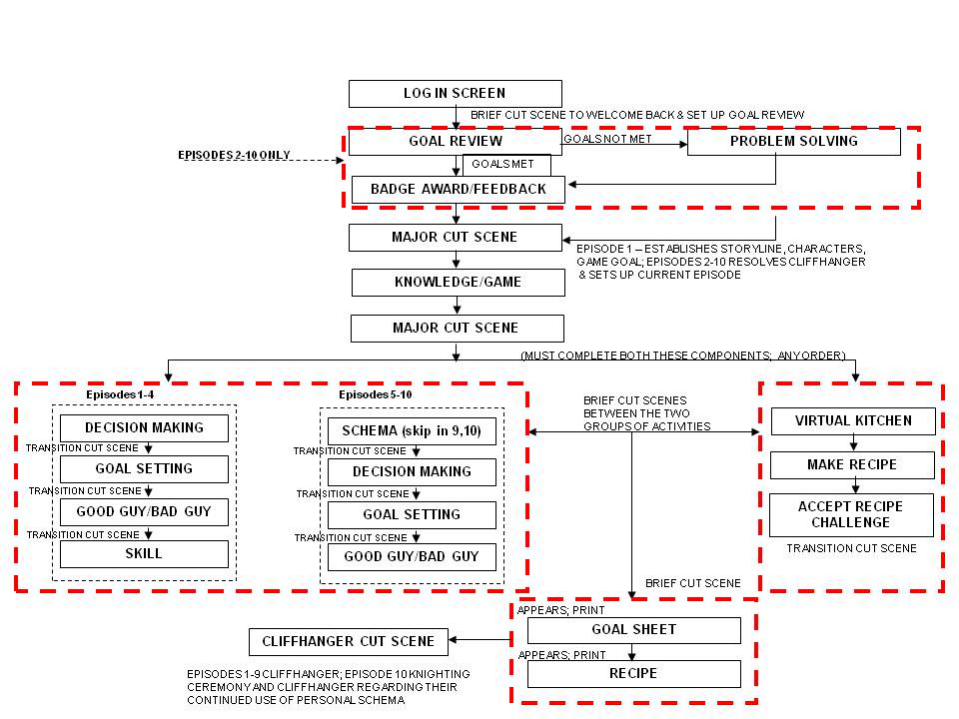
Game flow diagram.

The genre was action adventure. The game contents and flow diagram (ie, the video game structure) are presented in Figure 4 and are briefly described below.

#### Login Screen

Players were assigned a unique username and password with which to login to the game. After completing baseline data collection, players were randomly assigned to one of four versions of the game. Their username automatically routed them to their assigned version. The only differences between the groups were the implementation intentions described in the goal review and goal setting sections below.

#### Goal Review and Problem Solving

 Goal review appeared in episodes 2-10. It occurred at the beginning of each episode and was led by the Wizard. Players reported whether they met the two types of goals set during the previous episode: a FV consumption goal and a FV recipe preparation goal. In the video game, goals were referred to as challenges to be consistent with the “quest” towards knighthood. Players in one of the three groups that created an implementation intention reported whether they followed it. Players, regardless of group assignment, also reported whether they used the skills learned in the video game (ie, self monitoring, problem solving, and asking/negotiation) to help them meet their goals. For each unmet goal, the player participated in a problem solving sequence to identify the problem that kept them from meeting their goal. Players received feedback statements (tailored to their level of success in meeting their goals and/or whether they used skills to help them meet their goals). Players were then asked whether the effort they put into eating FV was worth it (an assessment of satisfaction). To reinforce goal commitment, they were then encouraged to type in a positive self statement (eg, “Setting and meeting my challenges shows I’ve got what it takes to be a winner!”) and read it out loud with conviction.

#### Badges

**Figure 5:**
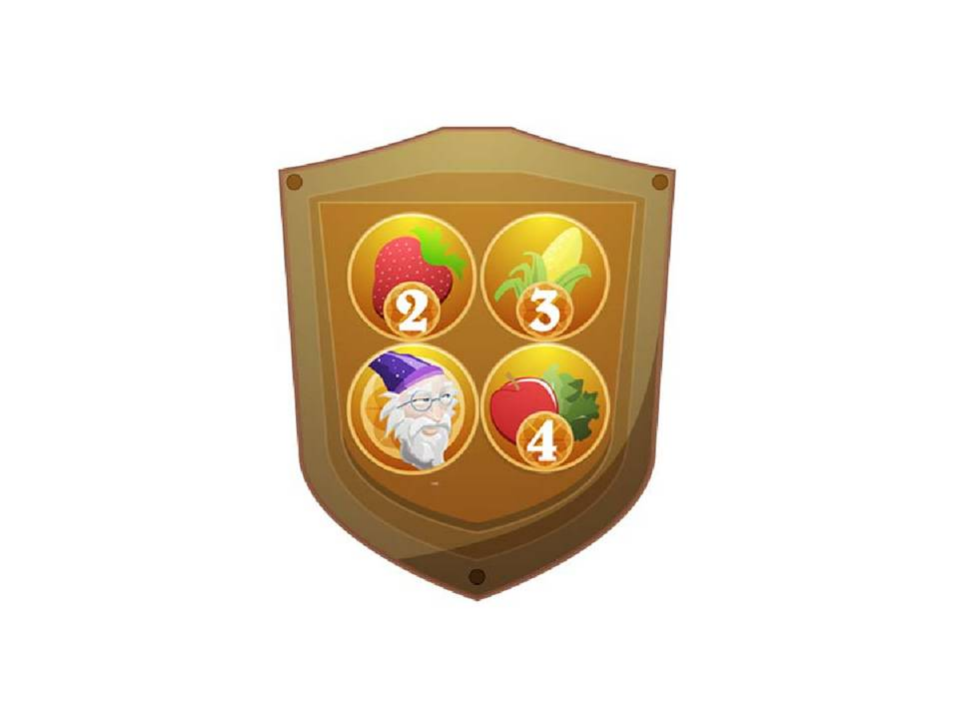
Shield with badges.

Badges were awarded in episodes 2 - 10 for meeting the goals set in the previous episode. Players could earn up to two badges each episode, one for meeting the FV goal and one for meeting the FV recipe goal. Total number of badges earned determined level of knighthood. There were five levels, ranging from the lowly Honorary Knight to the coveted Platinum Knight. Badges appeared on the player’s coat of arms (or shield) displayed in the castle foyer (Figure 5).

#### Cut Scenes

The cut scenes were animated video clips that presented the story. The story was told from the second person perspective. The cut scenes provided an opportunity for the characters to serve as role models through dialogue and action. To make the player feel “part of the action”, the characters “spoke” to the player by referring to him/her as “Squire”.

#### Knowledge + Mini-Game

**Figure 6:**
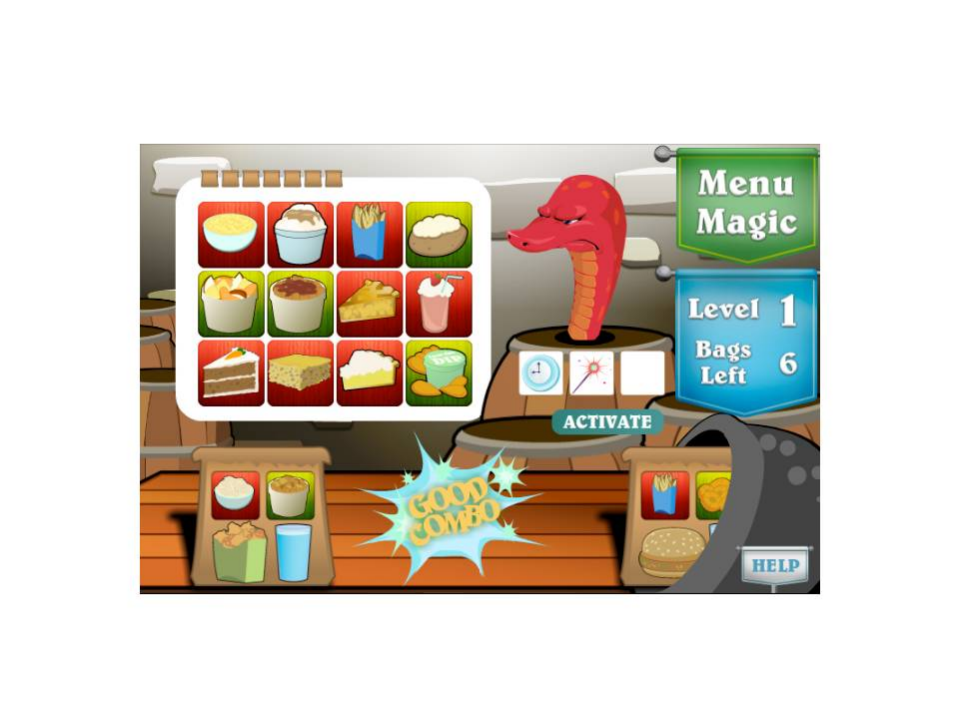
Mini-game screen shot.

This component appeared in episodes 1 - 10. It presented basic knowledge [24] about fruit, 100% juices, and vegetables (Table 1) and then reviewed, refined, and reinforced it in a timed “mini-game” (Table 2). The knowledge provided information the player needed to successfully complete the episode. Mini-games (Figure 6) contained progressively more difficult levels to promote mastery learning [28]. Game characters led this component.

**Table 1:** Knowledge topics.

Episode	Topic
1	100% fruit juices vs imposters; juice portion size
2	Real fruit vs fruit imposters
3	Real vegetables vs vegetable imposters
4	Recipe substitutions
5	Reinforcing portion size; memory joggers (eg, baseball, tennis ball)
6	FV for breakfast; breakfast on the go
7	FV in fast food restaurants
8	FV vs non-FV
9	Identifying number of FV in recipes
10	Review

**Table 2:** Mini-game descriptions.

Episode	Game	Description
1	100% Fruit Juice	The player creates chains by linking together 100% juices, 100% juice blends, or Not Juices.
2	Find the Fruit	From the various foods floating in the bubbles, the player must pop the bubbles with the fruits. The player must also identify fruit imposters – ie, high fat items containing some or no fruit.
3	Find the Veggies	From the various foods floating in the bubbles, the player must pop the bubbles with the veggies. The player must also identify veggie imposters – ie, high fat items containing some or no veggies.
4	Lunch-a-Bunch	The player must add fruit and vegetable items to the passing lunch trays to create lunches with 2 servings of fruit and/or vegetables.
5	The Mole Pole 1	A trivia game which tests the player’s knowledge of the information presented this episode; each correct answer allows them to progress through a tunnel. The goal is to exit the tunnel and get past the moles that guard it.
6	Breakfast Blunder	The player must add possible breakfast fruit and vegetable items to the food trays to create a breakfast with 2 servings of fruit and vegetables.
7	Fast Food Frenzy	The player must find possible fruit and vegetable choices on the menus of 3 different types of restaurants.
8	The Good Stuff	From the various foods floating in the bubbles, the player must pop the bubbles with fruit and vegetables.
9	The Mole Pole 2	A trivia game which tests the player’s knowledge of the information presented this episode; each correct answer allows them to progress through a tunnel. The goal is to exit the tunnel and get past the moles that guard it.
10	The Mole Pole Review	This trivia game is structured similarly to the others; the difference is it tests knowledge presented from all previous episodes.

#### Decision Making

Decision making, led by the Wizard, occurred in episodes 1 - 10. In episode 1, players selected their top three personal values; for each value, they then selected “reason statements” that linked meeting their FV goals with each personal value they selected [29]. For example, if a player chose “being successful” as one of their personal values, they were presented with the following reason statements: “Meeting my FV challenge” (ie, FV goal); “shows I can meet my challenges”; “shows I can make hard decisions and stick to them”; and “shows I work hard for what I want”. Each episode, the Wizard identified their goal (ie, challenge) (eg, “to eat fruit for snack”), then asked: “Which of these is a good reason to [insert challenge]?” The three values and corresponding reason statements the player selected in episode 1 were then presented for selection. This component was guided by Self Determination Theory, particularly the basic psychological need of “relatedness” - ie, one’s sense of connection [25].

#### Goal Setting

Goal setting, led by the Wizard, appeared in each episode. This component was tailored and interactive. In episode 1, players selected their favorite FV. In each episode, they were then presented with their favorite FV and decided which ones to use to meet their goal; they also selected the day(s) they would meet their goal. The FV goals became more difficult as the game progressed (Table 3). The groups varied only on whether they created an implementation intention during goal setting (eg, group 1 created no implementation intentions; group 2 created action intentions; group 3 created coping intentions; group 4 created both action and coping intentions).

**Table 3:** FV goals per episode.

Episode	Food	When	# of Days
1	F	Breakfast	1
2	F	Snack	1
3	V	Lunch	1
4	F or V	Dinner	1
5	V	Snack	2
6	F	Breakfast & Snack	2
7	V	Lunch & Dinner	2
8	FV schema	All day	3
9	FV schema	All day	3
10	FV schema	All day	daily

#### Behavioral Inoculation

Led by King Brocwell (the Good Guy) and King Sssynster (the Bad Guy), this component strengthened the player’s resistance to potential temptations [27]. King Brocwell supported the player’s decision to meet their goal and identified a potential temptation (ie, friends). King Sssynster then tried to tempt the player to not achieve their goal. King Brocwell refuted the temptation by reminding the player why meeting the goal was important to them (ie, the value-reason statement they selected in decision making).

#### Skills

This component appeared in episodes 1 – 4 to teach self-regulatory skills (ie, self monitoring, problem solving, asking/negotiation). This component was led by the game characters. Skills were taught through character modeling and dialogue.

#### Schemas

Schemas are guides for complex behavior [30-31]. In the video game, schemas were presented in episodes 5 - 8 to demonstrate various ways in which to consume 5 servings of FV a day. In these episodes, characters (Knights Julie and Alex, King, Queen, Wizard) each presented their schema (Table 4) then created a sample menu to demonstrate how they used it to plan their meals/snacks each day. The character then asked the player to locate the FV in the sample menu.

**Table 4:** Schemas.

Character	Schema
Knight Julie	1B, 1L, 1D, 2S
Knight Alex	2B, 1L, 1D, 1S
King	1B, 0L, 2D, 2S
Queen	1B, 2L, 1D, 1S
Wizard	1B, 1L, 2D, 1S

#### Virtual Kitchen

The virtual kitchen appeared in all ten episodes. It taught food preparation skills, planning, sequencing, and kitchen safety and promoted parent involvement. This component was interactive and included pre-steps involved in recipe preparation (ie, asking for permission, washing hands, etc) as well as a “virtual preparation” of the recipe (ie, a video clip that demonstrated how to prepare the recipe). With the exception of episode 1, players had a choice of recipes to prepare (Table 5). Recipes were selected from the Knight-in-Training cookbook. The robot, M.I.CH.A.E.L., guided the player through the Virtual Kitchen. At the end of this component, the player selected one of the recipes presented in each episode and set a goal to make the recipe at home.

**Table 5:** Recipes by episode.

Episode	Recipe Type	Recipe 1	Recipe 2	Recipe 3
1	Juice	Razzle Dazzle Juicy Delight	n/a	n/a
2	Fruit	On-the-Run Trail Mix	Fantastic Fruit & Chocolate	n/a
3	Vegetable	Fiery’s Black Bean Burrito	M.I.C.H.A.E.L.’S Veggie Wrap	n/a
4	Fruit & Vegetable	Fivealot’s Famous Fruit Salad	Knight Brocwell’s Stuffed Potatoes	n/a
5	Vegetable	Knight Julie’s Veggie Snack	Fiery’s Bean Dip	n/a
6	Fruit	Royal Smoothie	Squire’s Strawberry Split	n/a
7	Fruit & Vegetable	Power Pudding Dip	Wizard’s Magic Pocket	n/a
8	Fruit & Vegetable	Celebration Sundae	Chef Supremo’s Cinnamon Carrots	n/a
9	Fruit & Vegetable	Queen Nutritia’s Dip	Platinum Sweet Potatoes	n/a
10	Fruit & Vegetable	Knight Alex’s Banana Pops	Golden Knight Burrito	Moledred’s Ice Pops

### Parent Component

The parent component consisted of newsletters and access to a parent website. Each is briefly described below.

#### Newsletters

**Figure 7:**
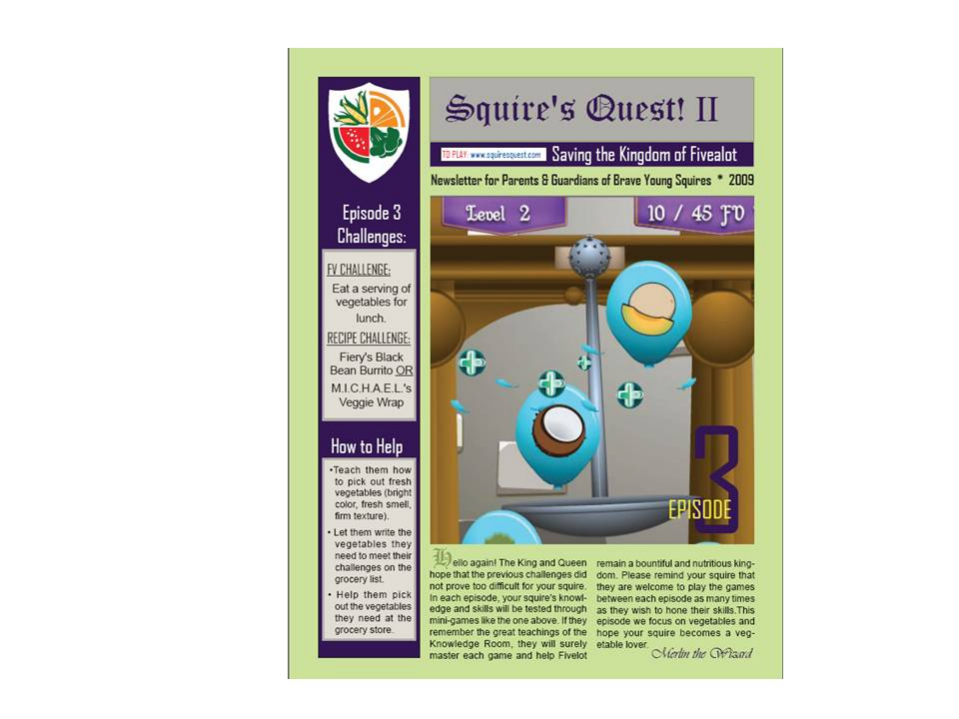
Parent newsletter.

Parent newsletters were designed to promote parent involvement (Figure 7). There were ten newsletters – one matched to each episode of the video game. Newsletters were emailed to parents prior to each episode of the video game. Each newsletter identified the child’s general FV and recipe goals for the upcoming episode and provided tips for what the parent could do to help their child meet their goals. Each newsletter also identified vocabulary words (ie, words used in video game with which the child may not be familiar, such as “ingredients”) and provided healthy FV recipes and suggestions for overcoming common FV problems families face when attempting to eat FV (Table 6).

**Table 6:** Parent newsletter.

Episode	Focus	Recipe
1	100% Fruit Juice	Peach Cobbler
2	Added Sugar in Fruits	Strawberry Shortcake
3	Vegetables	Black Bean Soup
4	Fruit and Vegetable Substitutions	Vegetable Lasagna
5	Serving Size Review	Tomato and Bean Dip
6	Vegetables for Breakfast	Breakfast Potatoes
7	Eating Out	Hearty Rice
8	Empty Calories	Round Table Pizza
9	Serving Size Comparisons	Vegetable Soup
10	Final Tips	Blueberry Dessert Cups

#### Website

The parent website provided information to create a healthy home nutrition and activity environment. It included family-friendly recipes and addressed topics such as grocery shopping, eating on the go, and getting the family involved in physical activity. Information was routinely updated (ie, ten updates corresponding to the ten-episode video game) (Table 7).

**Table 7:** Parent website.

Episode	Focus	Recipes
1	Shopping Lists & A Well-Stocked Kitchen	Baked apples, cinnamon roasted sweet potatoes, spinach and strawberry salad
2	Getting Kids Involved in the Kitchen	Vegetable pasta, baked bananas, veggie grilled cheese
3	Family Meals	Spanish paella, gazpacho, and strawberry flurry
4	Family Activity Time	Bran muffins, vegetable omelet, fruit parfait
5	Buying & Storing Food	Chicken salad sandwich
6	Food & Kitchen Safety	Veggie couscous, turkey, light fish, pineapple orange frozen yogurt
7	Nutrition Facts Labels	Slow cooker chicken, three bean chili, tossed salad, potato bake
8	Portion Control	Layered salad, brown rice casserole, fruit salad, and chocolate berry cake
9	Eating on the Go	Fruit and nut mix, hummus with veggies, chicken salad sandwich
10	Substitutions	Broccoli mac-and-cheese, baked chicken nuggets, black bean brownies, pineapple angel food cake

### Procedures

#### Intervention

When children were eligible to play the next episode of the video game, an email with a link to the login page was automatically generated and sent to them. Simultaneously, parents received emails with links to the online newsletters and the parent website. An access database tracked participants (parents, children) through the program. Alerts notified the research team when parents and children were eligible to receive the next intervention component (ie, next game episode; next newsletter) and when they were eligible for data collection. The video game was programmed to notify the intervention staff when the child completed an episode, and emails were automatically generated when a parent opened the newsletter email or when the parent website was accessed. If the child did not log on to play the next episode of the game within approximately five days, they were contacted by the intervention staff. The intervention research team was available by email or phone to provide technical assistance. As part of process evaluation, all participant contacts were recorded in the access database.

#### Data Collection

To assess usual dietary intake, three unannounced 24-hour dietary recalls were obtained at each data collection period using the Nutrient Data System for Research (NDSR-2009), University of Minnesota [32]. The 24-hour dietary recalls were conducted directly with the child; two weekday and one weekend day recalls were obtained using a laptop computer, NDSR-2009 software [33], and 2-dimensional food and measurement models. A paper copy of the models was given to the family for use in the telephone interviews. The child was asked where each meal/snack was eaten, who else was present, whether a TV was on, and whether they watched TV during the meal. The dietary recalls were analyzed for servings of FV [34].

Child psychosocial characteristics (FV preferences [35], asking behaviors [36], self efficacy [37], and outcome expectancies [38]) were collected using existing measures, some of which were adapted for this study. Using the work of Rothman [20] and Green and Brock [39] respectively, child satisfaction and game immersion were assessed using scales developed for this study. Social desirability [40-41] was also collected to control for potential bias in self report data. Brief, semi-structured interviews were conducted with children to further assess their reactions to the game. Parents provided self report data (parent FV consumption [42], home FV availability [43], home FV accessibility [36, 44], family barriers to eating FV [45], parent self efficacy to get their family to eat FV [45], child FV asking behaviors [36], and child executive function [46]). In addition, they provided demographic information at baseline. Self-report data were collected online over a secure, password protected website. Parents and children were each provided unique passwords with which to log on to the data collection website.

 Following the framework of Baranowski and Jago [47], process data were collected through staff logs, as children navigated the game, and as parents accessed the parent components. Examples of process data included: recruitment of participants, maintenance of participation, implementation (fidelity and extent), implementation barriers, program exposure, initial use of program, continued use of the program, and contamination. Implementation and exposure assessments were documented using electronic logon records. Game-play data (Figure 4) were collected as children played each episode (eg, logons, goals set, goals attained, values and reasons, number of badges, recipes selected, action intentions, coping intentions). Email open rate (parent newsletters) and visits to the parent website were also collected. Self-report appeal and use of intervention components were collected from children and parents at post 1 data collection (Table 8).

**Table 8:** Measures

Who	How	What	Baseline	During	Post 1^a^	Post 2^b^
Child	Phone	FV intake (3, 24hr DR ^c^)	x		x	x
	Online	FV Preferences	x		x	x
		FV Asking Behaviors	x		x	x
		FV Self Efficacy	x		x	x
		FV Outcome Expectations	x		x	x
		Satisfaction With Change		x	x	x
		Immersion			x	
		Social Desirability	x			
		Game Likability			x	
	Gameplay	Logons		x		
		Responses/Choices (ie, goals set/attained, values/reasons, etc)		x		
	Interview	Game Reactions			x	x
Parent	Online	FV Intake (self)	x		x	x
		Home FV Availability	x		x	x
		Home FV Accessibility	x		x	x
		Family Barriers	x		x	x
		Self Efficacy	x		x	x
		Child Asking Behaviors	x		x	x
		Child Executive Function	x		x	x
		Demographic Information	x			
		Overall Reactions/Use			x	
		Email Open Rate		x		
		Website Visits		x		
Staff	Logs	Process Evaluation	x	x	x	x

^a^3 months after baseline assessment

^b^6 months after baseline assessment

^c^dietary recall

#### Data Analyses

Repeated measures analyses of variance/covariance, controlling for key demographic factors, baseline FV consumption, and energy intake, accommodated a two-level within factor (post 1, post 2) and a four-level between-groups factor design. The group’s main effect allowed investigation of group differences, regardless of whether it was post 1 or post 2. The group-by-time interaction term allowed investigation of group differences over time, thus identifying if the treatment was maintained. Univariate outcomes (the number of goals achieved, number of newsletters read, etc) were analyzed using a univariate one between-group factor design. Secondary analyses included investigation of trends in goal attainment, FV consumption, and psychosocial factors across study weeks through the use of Chi-square analyses for ordinal repeated measures. A dose-response analysis was also planned.

## Discussion

This report provides a description of the protocol, procedures, and assessment tools for a video game designed to increase FV consumption among children. This research has several strengths. First, it is based on an earlier video game that successfully increased FV consumption in 4^th^ grade children [10]; it was designed within a multi-theoretical framework; and it systematically varies only one component, implementation intentions. Strengths also include a large sample size, a focus on both parents and children, and examination of maintenance effects. Finally, it uses a strong measure of dietary data collection (3, 24 hour dietary recalls at each data collection period). However, this study is conducted in one specific geographic region, thus limiting its generalizability.

To our knowledge, this is the first study to test the effect of implementation intentions on FV goal attainment and consumption and to examine the relationship between satisfaction and maintenance of behavior change in pre-adolescents. The intervention includes a parent and child component designed within an integrated theoretical framework to maximize the likelihood of behavior change. The successful implementation of this intervention will generate valuable information regarding the effectiveness of this approach for young children.

## Abbreviations

B: Breakfast
FV: Fruit(s), vegetable(s)
L: Lunch
D: Dinner
N/A: Not applicable
S: Snack
SQ!2: Squire’s Quest! II: Saving the Kingdom of Fivealot
